# SOCIODEMOGRAPHIC AND HEALTH DISPARITIES IN USE OF PREVENTIVE HEALTHCARE SERVICES

**DOI:** 10.1093/geroni/igz038.3201

**Published:** 2019-11-08

**Authors:** Terese Sara Hoej Joergensen, Heather Allore, Corey Nagel, Ana R Quiñones

**Affiliations:** 1 Oregon Health and Science University, Portland, United States; 2 Yale University, New Haven, Connecticut, United States; 3 University of Arkansas for Medical Sciences, Little Rock, Arkansas, United States; 4 Family Medicine, Oregon Health & Science University, Portland, Oregon, United States

## Abstract

Introduction: Older adults experience multiple health problems necessitating medical care. However, studies have shown that healthcare is not equally accessible for older adults in the US. In 2011, Medicare introduced Annual Wellness Visits to improve access to preventive healthcare including influenza vaccination. Objectives: Ascertain whether sociodemographic factors, multimorbidity, and other health indicators pose a barrier for older adults to access Annual Wellness Visits and influenza vaccination. Methods: We analyzed data from the 2012 and 2014 waves of the Health and Retirement Study linked with Medicare records. 4,858 older adults aged 65+ years were included in Conditional Random Forests to identify the most important predictors of Annual Wellness Visits and influenza vaccination during this two-year period. The predictors included: age, sex, race/ethnicity, partnered, geographical region, wealth, educational level, Medicaid coverage, body mass index, activities and instrumental activities of daily living, proxy interview, cognitive impairment, dementia diagnosis, and multimorbidity. Results: In total, 1,142 (23.6%) older adults had an Annual Wellness Visit and 3,316 (68.4%) older adults received an influenza vaccination. 11.9% were non-Hispanic black, 6.3% were Hispanic, with a median of 6 chronic conditions and 16.9% had dementia. The most important predictors of Annual Wellness Visits were region, wealth, dementia diagnosis, and race/ethnicity. The most important predictors of influenza vaccination were multimorbidity, race/ethnicity, educational level, and wealth. Conclusion: The importance of geographical region for Annual Wellness Visits suggests that the service has not been adopted equally throughout the US, whereas multimorbidity is the most important factor for receiving influenza vaccination.

